# Tetratricopeptide repeat domain 3 overexpression tends to form aggregates and inhibit ubiquitination and degradation of DNA polymerase γ

**DOI:** 10.18632/oncotarget.22476

**Published:** 2017-11-17

**Authors:** Yueqing Gong, Xiaolan Wang, Xuan Shang, Sheng Ping Xiao, Wanjie Li, Yu Shang, Fei Dou

**Affiliations:** ^1^ State Key Laboratory of Cognitive Neuroscience and Learning and IDG, McGovern Institute for Brain Research, College of Life Sciences, Beijing Normal University, Beijing, China; ^2^ Key Laboratory of Cell Proliferation and Regulation Biology, Ministry of Education, College of Life Sciences, Beijing Normal University, Beijing, China; ^3^ Center for Collaboration and Innovation in Brain and Learning Sciences, Beijing Normal University, Beijing, China

**Keywords:** TTC3, POLG, ubiquitination, proteostasis, mitochondrion

## Abstract

Tetratricopeptide repeat (TPR) domain 3 (TTC3) is a protein that contains canonical RING finger and TPR motifs. It is encoded by the *TTC3* gene located in the Down syndrome critical region (DSCR). In this study, we used a yeast two-hybrid assay to identify several proteins that physically interact with TTC3, including heat shock proteins and DNA polymerase γ (POLG). When TTC3 was overexpressed in mammalian cells, the ubiquitination of POLG was inhibited and its degradation slowed. High TTC3 protein expression led to the development of intracellular TTC3 aggregates, which also contained POLG. Co-expression with Hsp70 or placing the TTC3 gene under control of an inducible promoter alleviated the aggregation and facilitated POLG degradation. As a result of POLG’s effects on aging processes, we propose that a copy number variant of the *TTC3* may contribute to Down syndrome pathogenesis.

## INTRODUCTION

Human tetratricopeptide repeat (TPR) domain 3 (*TTC3*) is a gene located on chromosome 21q22.2 within the Down syndrome (DS) critical region (DSCR); it encodes a protein of 2025 amino acid residues [1, 2]. DSCR is a region covering 21q11.2 and 21q22.1–22.3, which are thought to be associated with most DS features [3–5]. Northern blot analyses have shown that TTC3 expression is regulated, to some extent, in a tissue-specific manner [1, 2, 6]. Further research has shown that TTC3 expression is developmentally regulated during human and mouse embryogenesis [7, 8]. At the earliest stages of development, TTC3 expression is ubiquitous. At later developmental stages, it becomes restricted to the nervous system.

TTC3 protein harbors a RING finger domain and pairs of TPR motifs [9, 10]. The TPR motif is a protein–protein interaction module of 34 amino acids. It is found in multiple copies of a number of functionally different proteins that have specific interactions with partner proteins [11], such as the interactions between chaperone and its cofactors (reviewed in McClellan *et al.* [12]). The conserved domain pattern of TTC3 suggests that it could be a co-chaperone, linking chaperones and the ubiquitin-proteasome system and participating in the maintenance of protein homeostasis.

Few reports exist on the physiologic functions of TTC3. Previous studies have shown that TTC3 affects cell proliferation and differentiation. TTC3 inhibits neuronal differentiation via RhoA and citron kinase [13]. In addition, TTC3 is an E3 ligase that binds phosphorylated Akt and can silence its activity via a proteasomal cascade. As a result, overexpression of TTC3 leads to a significant accumulation of G2/M cells and inhibits cell proliferation [9].

In this study, we identified an interacting partner of TTC3, DNA polymerase subunit gamma-1 (encoded by the POLG gene), using yeast two-hybrid systems with human TTC3 (aa 1-650) as bait. POLG protein is the catalytic subunit of DNA polymerase γ (pol γ), which is the only polymerase found in animal cell mitochondria. In addition, we found that TTC3 interacts with Hsp70, as expected. Our results showed that TTC3 overexpression forms aggregate structures in cells and slows the degradation of POLG. Co-expression with Hsp70 or placing the TTC3 gene under control of an inducible promoter alleviates the aggregation. Under these conditions, the soluble form of TTC3 facilitates the poly-ubiquitination of the POLG protein and accelerates its degradation. These findings suggest that TTC3 could be a potential E3 ligase for POLG and involved in its degradation. As a result of its interaction with POLG, TTC3 might participate in the maintenance of mitochondrial functions, and mutation or copy number variant of the TTC3 gene may contribute to the pathogenesis of different neurodegenerative diseases, such as DS and Alzheimer disease.

## RESULTS

### TTC3 proteins interact with POLG and Hsp70

**Figure 1:**
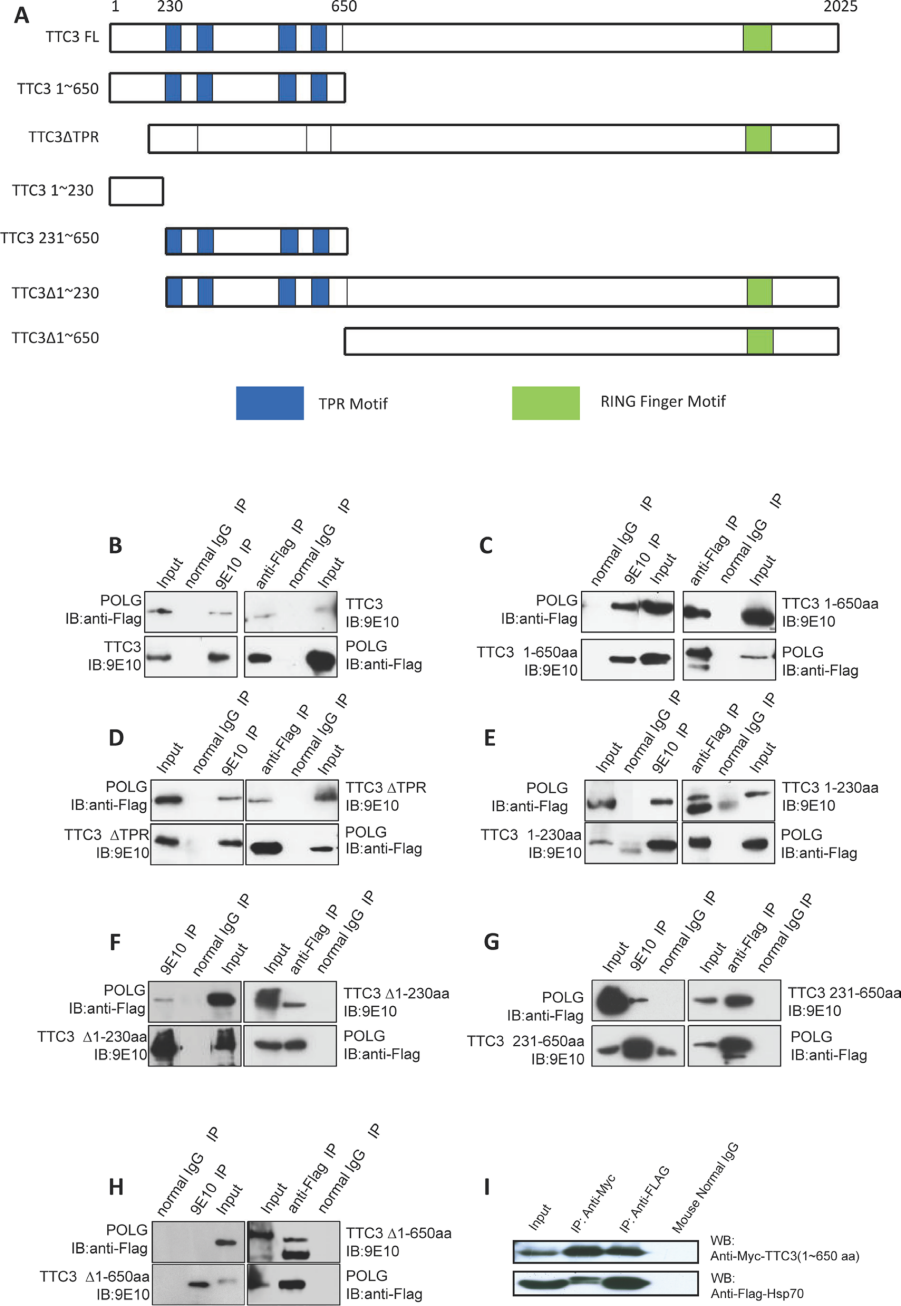
TTC3 proteins interact with POLG and Hsp70 (**A**) Diagram shows the structure and functional domains of Myc-tagged wild-type TTC3 and subfragments of TTC3 in mammalian expression vectors (pCMV-Myc) used in our study. (**B** to **H**) Co-immunoprecipitation experiment. Myc-tagged TTC3 (WT-TTC3 or its subfragments) and 3xFlag-tagged POLG were transfected into 293T cells. TTC3 was immunoprecipitated by Myc antibody (9E10), and POLG was immunoprecipitated by Flag antibody (M2). (**I**) Co-immunoprecipitation experiment. Myc-TTC3(1∼650aa) and Flag-Hsp70 were transfected into 293T cells. TTC3 was immunoprecipitated by Myc antibody (9E10), and Hsp70 was immunoprecipitated by Flag antibody (M2).

A stable interaction between TTC3 and POLG was found by using yeast two-hybrid systems with human TTC3 (aa 1-650) as bait. The interaction between full-length TTC3 and POLG was confirmed in a co-immunoprecipitation experiment (Figure 1B, 1C). Next, we constructed vectors expressing a series of truncated TTC3 fragments to further define the interacting domains with POLG (Figure 1A). Co-immunoprecipitation experiments showed that TTC3Δ650 (TTC3 that lacks the N-terminal 1∼650 residues) could not interact with full-length POLG (Figure 1H). The N-terminal 1∼650 residues harbor TPR motifs (Figure 1A). Independent deletion of N-terminal 1∼230 residues (Δ230) or the TPR motifs (ΔTPR) did not completely block the interaction between TTC3 and full-length POLG (Figure 1D, 1F), and both of the N-terminal fragments of 1∼230 residues and 231∼650 residues were able to interact with POLG (Figure 1E, 1G). The 1∼230 residue fragment showed a stronger interaction with POLG than the 231∼650 residue fragment. These results suggest that the interaction between TTC3 and POLG is initiated through the N-terminal 1∼650 residues, with the N-terminal 1∼230 residues being most important. In addition, yeast two-hybrid systems showed that TTC3 interacts with Hsp70. This interaction was also confirmed in a co-immunoprecipitation experiment (Figure 1I).

### TTC3 overexpression decreases POLG ubiquitination and slows its degradation

**Figure 2:**
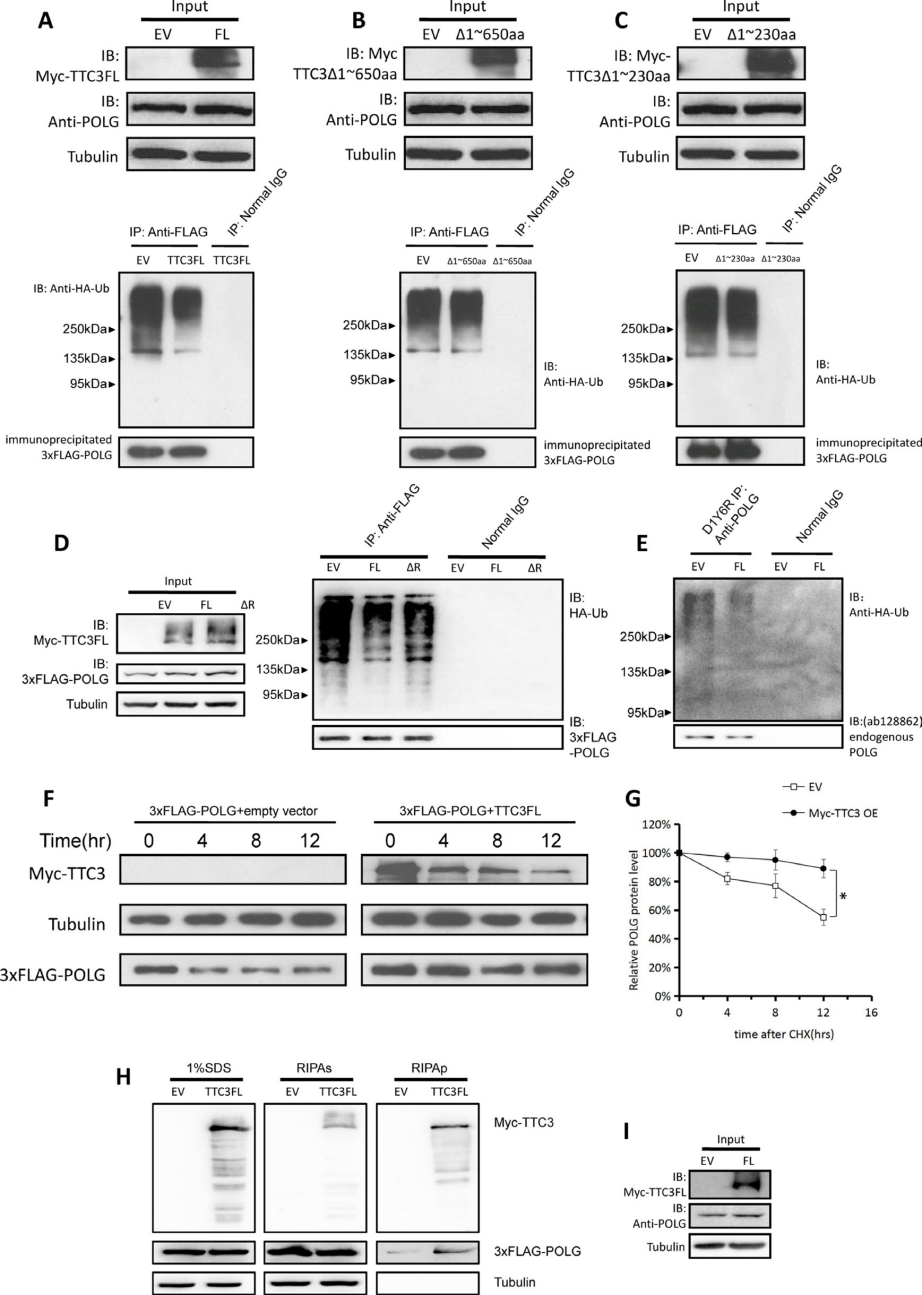
Overexpression of TTC3 slows degradation of POLG (**A**) Wild-type TTC3, 3xFLAG-POLG, and HA-Ub were transfected into 293T cells. TTC3 overexpression resulted in a lower level of POLG poly-ubiquitination than the control vector (pCMV-Myc empty vector). (**B** and **C**) Because the interaction between TTC3 and POLG was mediated through the N-terminal 1∼650 residues and predominantly through the N-terminal 1∼230 residues, we performed the same protocol with Δ650 (B) and Δ230 (C). With Δ650, the poly-ubiquitination of POLG was not significantly lower than in the control vector. With Δ230, the poly-ubiquitination of POLG was slightly lower than in the control vector. (**D**) TTC3ΔRING had similar effects on POLG ubiquitination (right panel), which had a consistent level with TTC3FL (left panel, input). (**E** and **I**) Endogenous POLG ubiquitination with TTC3 overexpression. Wild-type TTC3 or pCMV-Myc empty vector was transfected into 293T cells with HA-Ub. TTC3 overexpression resulted in a lower level of endogenous POLG poly-ubiquitination than in the control vector (pCMV-Myc empty vector). The inputs are shown in panel I. (**F** and **G**) CHX assay. 293T cells were transfected with wild-type TTC3 and 3xFLAG-POLG. TTC3 slowed down the degradation of POLG compared to the control vector. Relative intensities were measured using NIH Image J (G). POLG band intensity was normalized to α-tubulin, and then normalized to the 0 hour time point. The error bars represent mean ± standard error (SE) (*n* = 3). ^*^P < 0.05. (**H**) Myc-tagged TTC3 (WT-TTC3 or its subfragments) and 3xFlag-tagged POLG were transfected into 293T cells. The cells were harvested 24 hours after transfection and lysed with RIPA buffer for the RIPA-soluble and -insoluble parts. TTC3 increased the POLG level in RIPA-insoluble part, suggesting that TTC3 overexpression could bring POLG protein into aggregates.

The *TTC3* gene encodes a canonical E3 RING finger motif at the C-terminus. Previous studies showed that TTC3 induces poly-ubiquitination of Akt and facilitates its proteasomal degradation [9]. In light of the interaction between TTC3 and POLG, we attempted to determine whether TTC3 has the same effect on POLG as on Akt. To investigate the effect of TTC3 overexpression on the ubiquitination of POLG protein, we transfected wild-type TTC3 (or the control empty vector), exogenous 3xFLAG-POLG, and HA-tagged ubiquitin (HA-Ub) into 293T cells. Western blot analyses showed that TTC3 overexpression reduced POLG ubiquitination levels (Figure 2A). We also found that overexpression of TTC3ΔRING (TTC3 that lacks the RING finger motif) has the same effect (Figure 2D). This indicates that the decrease in POLG ubiquitination level is not associated with Ub ligase activity of TTC3. Moreover, the partially binding-defective mutant (Δ230) resulted in smaller decrease in POLG ubiquitination (Figure 2C), whereas expression of the binding-defective mutant (Δ650) did not change POLG ubiquitination (Figure 2B). These results suggest that the decrease of ubiquitination depends on the TTC3-POLG interaction. Consistently, TTC3 reduced the ubiquitination level of endogenous POLG (Figure 2E; inputs are shown in Figure 2I). In addition, to investigate POLG degradation, CHX was applied to inhibit protein biosynthesis. Western blot showed that TTC3 slows POLG degradation (Figure 2F, 2G).

### Overexpressed TTC3 forms insoluble aggregates in cells, and Hsp70 reduces these aggregates

**Figure 3:**
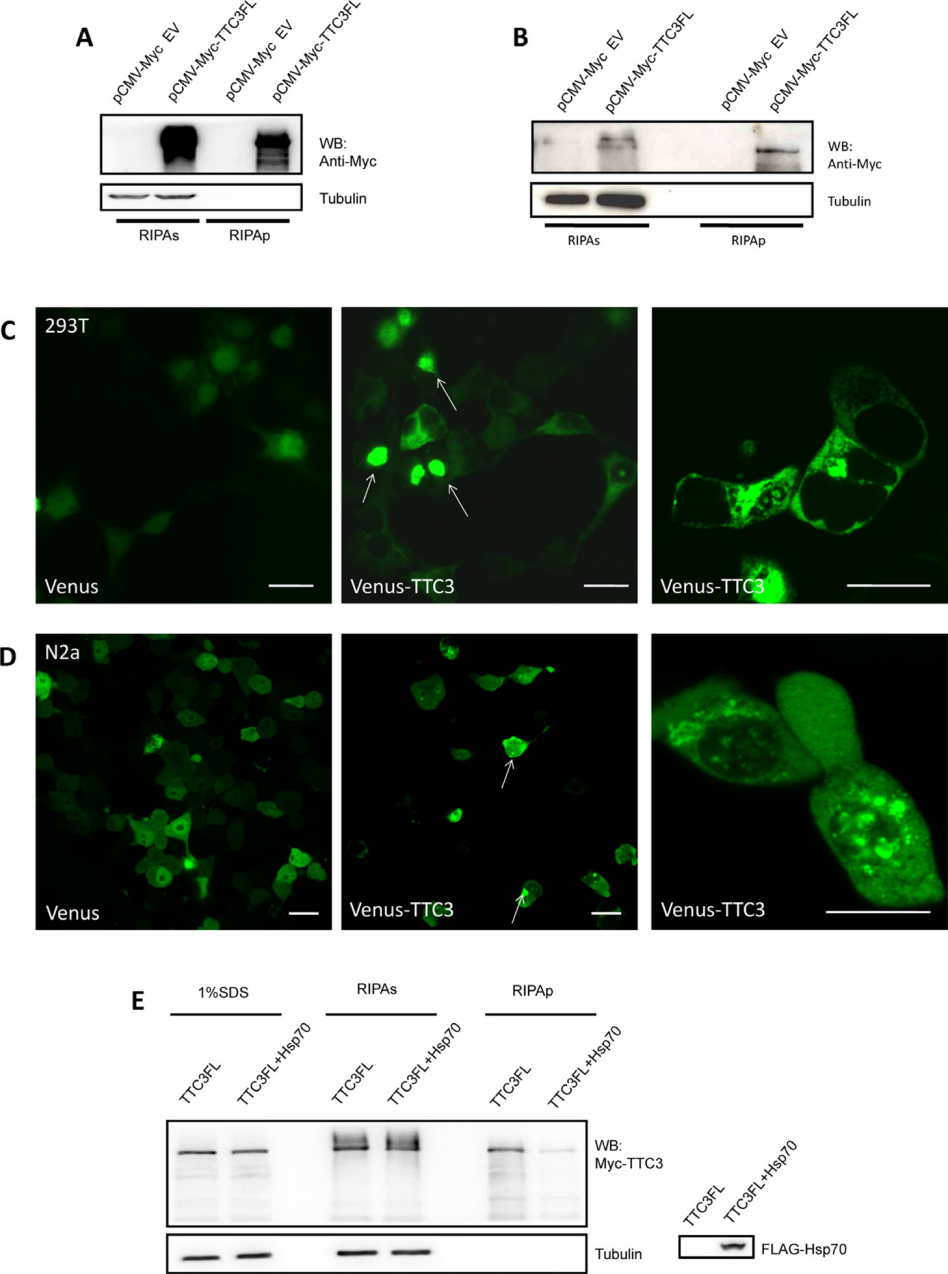
Overexpression of TTC3 forms aggregate structures in cells (**A** and **B**) TTC3 forms RIPA-insoluble components. 293T cells (A) and N2a cells (B) were transfected with pCMV-Myc-TTC3 by TurboFect reagents. The cells were harvested 24 hours after transfection and lysed with RIPA buffer for the RIPA-soluble and -insoluble parts. The RIPA lysates were clarified by centrifugation at 16,000 × *g* for 20 minutes, and the pellets were sonicated with an equal volume of SDS buffer and dissolved. TTC3 protein occurred in the RIPA-insoluble part. (**C** and **D**) 293T (C) and N2a (D) cells were transfected with Venus-TTC3FL (N-terminal Venus-fused TTC3) or Venus. Image recording was performed 48 hours after transfection. Scale bar = 20 μm. (**E**) Hsp70 improves solubility of TTC3. 293T cells were transfected with FLAG-Hsp70 and Myc-TTC3. Co-transfection with Hsp70 decreased the amount of RIPA-insoluble TTC3 proteins.

We further investigated how TTC3 inhibits POLG ubiquitination and degradation. Confocal microscopy was used to define the cellular localization of TTC3. Venus-fused TTC3 was expressed in 293T and N2a cells, and a significant number of cells formed TTC3 aggregates (Figure 3C, 3D). Western blot analyses confirmed these results. A sequential protein extraction method was used to isolate the proteins with different solubility. The harvested cells were lysed with RIPA buffer. After centrifugation, the RIPA-insoluble pellets were sonicated with buffer containing 1% SDS and dissolved. We found that TTC3 presented in the RIPA-insoluble part of cells (Figure 3A, 3B). Thus, overexpression of TTC3 proteins may cause the formation of nonfunctional aggregates and interrupt other potential pathways for POLG degradation. Next, we attempted to reduce the aggregation of TTC3, by improving its solubility, to restore the physiologic functions of the native spatial structure. We found that TTC3 solubility is improved by co-transfection with Hsp70 (Figure 3E).

It is worth noting that myc-TTC3 bands are higher in RIPAs lanes than in RIPAp lanes (shown in Figure 3A, 3B, and 3E). We believe two factors could contribute to this phenomenon. First, aggregation alters the posttranslational modification of TTC3 protein. The misfolded protein might not be modified correctly, so the TTC3 protein in the RIPA-soluble and -insoluble parts of cell lysis could have different molecular weights. Second, our previous research showed that TTC3 could be hydrolyzed into fragments in cells, which could lead to differences in molecular weight.

### Soluble TTC3 facilitates POLG degradation

**Figure 4:**
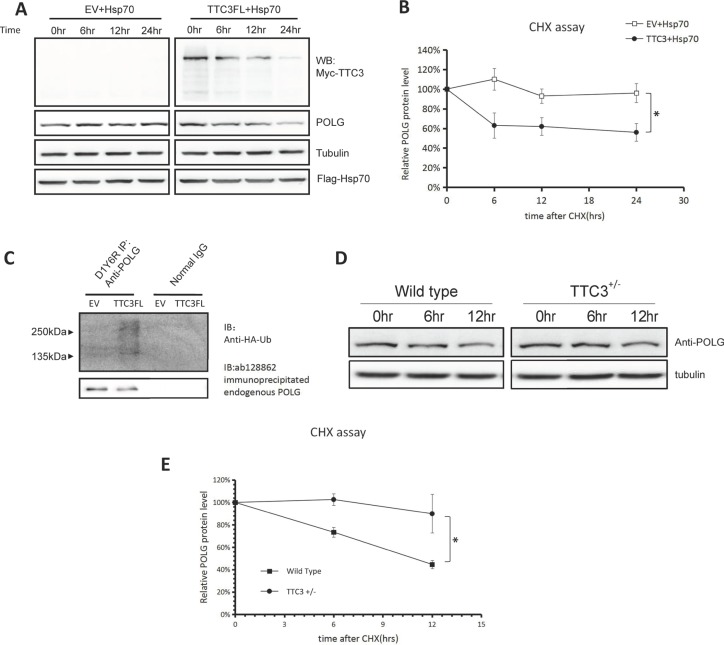
TTC3 facilitates POLG degradation in the presence of co-transfected Hsp70 (**A** and **B**) CHX assay. With co-transfected Hsp70, TTC3 increased the POLG degradation rate. Times (hours) are shown after CHX treatment (time 0). Relative intensities were measured using NIH Image J (B). POLG band intensity was normalized to α-tubulin and then normalized to the 0 hour time point. The error bars represent mean ± standard error (SE) (*n* = 3). ^*^P < 0.05. (**C**) Endogenous POLG ubiquitination with co-transfected TTC3 and Hsp70. Wild-type TTC3 or pCMV-Myc empty vector was transfected into 293T cells with FLAG-Hsp70 and HA-Ub and resulted in a higher level of endogenous POLG poly-ubiquitination than in the control vector (pCMV-Myc empty vector). (**D** and **E**) CHX assay. A reverse experiment knocking out one copy of endogenous TTC3 was performed. The POLG degradation rate was lower in the TTC3+/– MEFs than in the wild-type MEFs. Times (hours) are shown after CHX treatment (time 0). Relative intensities were measured using NIH Image J (E). POLG band intensity was normalized to α-tubulin, and then normalized to the 0 hour time point. The error bars represent mean ± SE (*n* = 3). ^*^P < 0.05.

We investigated whether soluble TTC3 has different effects on POLG. Using a CHX chase assay, we found that TTC3 increases the degradation rate of endogenous POLG with Hsp70 co-transfection (Figure 4A, 4B). TTC3 also increased the ubiquitination level of endogenous POLG in the presence of co-transfected Hsp70 (Figure 4C). Knocking out one copy of *TTC3* (*TTC3*+/–) also decreased the POLG degradation rate in mouse embryo fibroblasts (Figure 4D, 4E). Homozygous knockout of *TTC3* (*TTC3*–/–) is lethal, so we did not test homozygous cells.

**Figure 5:**
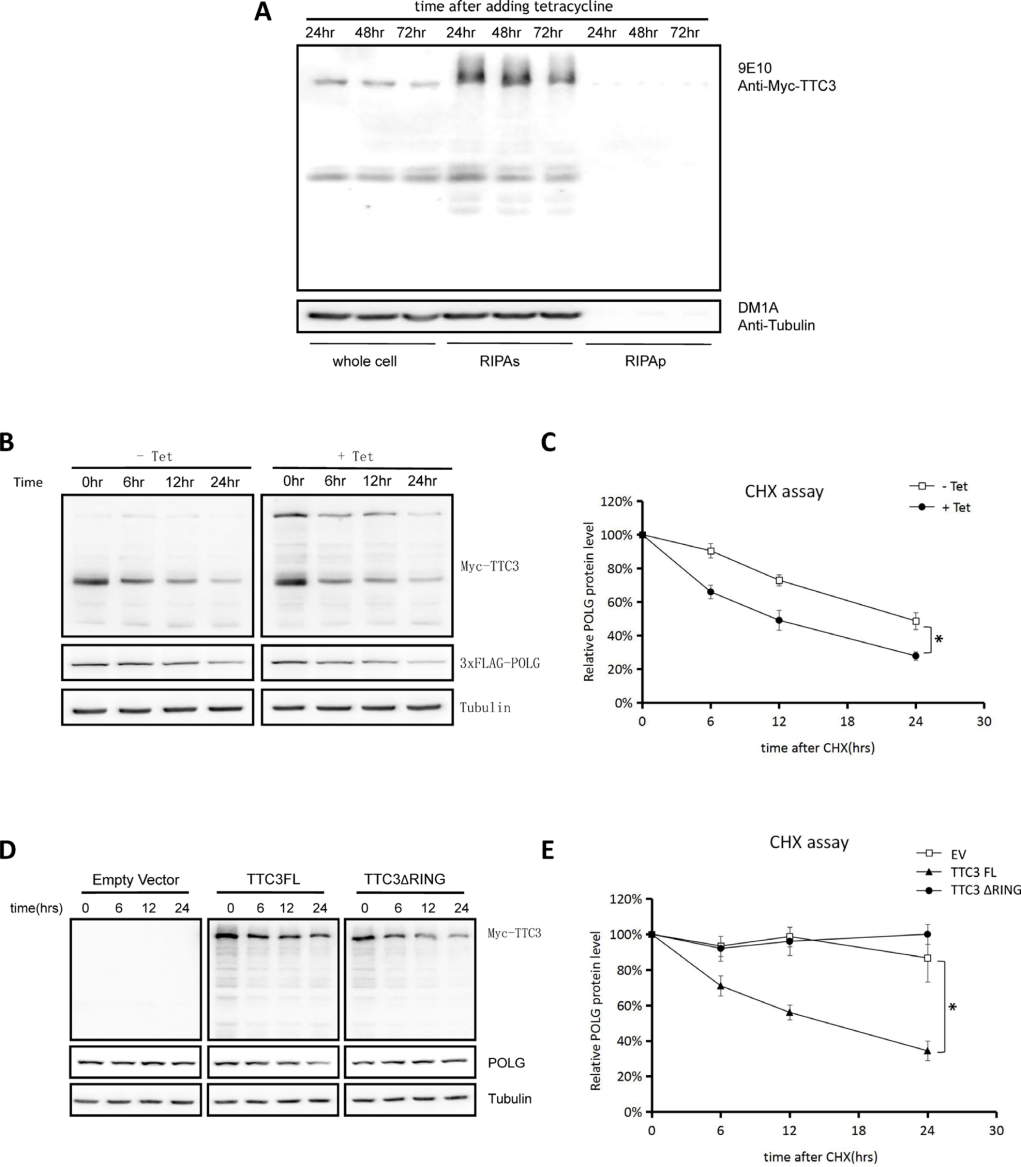
Soluble TTC3 facilitates POLG degradation (**A**) We constructed an Myc-TTC3 Tet-on 293T stable cell line. Cells were treated with 1 μg/mL of tetracycline for 24, 48, or 72 hours. The cells were harvested at indicated time points and lysed with SDS buffer for the whole-cell components or lysed with RIPA buffer for the RIPA-soluble and -insoluble parts. The RIPA lysates were clarified by centrifugation at 16,000 × *g* for 20 minutes, and the pellets were sonicated with an equal volume of SDS buffer and dissolved. The cells expressed Myc-TTC3 steadily and mildly, and TTC3 was primarily RIPA soluble. (**B** and **C**) CHX assay. Using a Tet-on stable cell strain, we obtained overexpression of soluble TTC3, which resulted in a faster degradation of POLG. Times (hours) are shown after CHX treatment (time 0). Relative intensities were measured using NIH Image J (C). POLG band intensity was normalized to α-tubulin and then normalized to the 0 hour time point. The error bars represent mean ± standard error (SE) (*n* = 3). ^*^P < 0.05. (**D** and **E**) CHX assay. By reducing the DNA amount of transfected TTC3 plasmids, we obtained overexpression of soluble TTC3, which resulted in faster degradation of POLG. Times (hours) are shown after CHX treatment (time 0). Relative intensities were measured using NIH Image J (E). POLG band intensity was normalized to α-tubulin and then normalized to the 0 hour time point. The error bars represent mean ± SE (*n* = 3). ^*^P < 0.05.

In addition to co-transfection with Hsp70, solubility of TTC3 was also improved by reducing TTC3 expression. To reduce TTC3 expression, we constructed an Myc-TTC3 stable cell line by using a Tet-on system, which resulted in the overexpression of soluble TTC3 (Figure 5A). We also reduced TTC3 overexpression by optimizing the transient transfection protocol. In both cases, the soluble form of TTC3 increased the POLG degradation rate (Figure 5B and 5C, with the stable cell line; Figure 5D and 5E, with optimized transfection protocol). In addition, wild-type TTC3, but not TTC3ΔRING, increased POLG degradation (Figure 5D and 5E), suggesting that TTC3 may act as an E3 ligase in this process.

## DISCUSSION

Chromosome 21 contains many genes that contribute to different phenotypes of DS (reviewed in Antonarakis *et al.* [14]). Although a complete extra copy of chromosome 21 is the most common cause of DS [14], extra chromosome 21 content can arise in a variety of ways and contribute to DS. This diversity of gene overexpression and functions leads to phenotypic diversity, which is why DS symptoms vary in patients. As a gene located in DSCR, TTC3 might contribute to certain phenotypes.

Recently, it has been suggested that TTC3 may play an important role in individual development. TTC3 is an E3 ligase that binds phosphorylated Akt and can silence its activity via a proteasomal cascade, and Akt is an intersection of multiple signaling pathways [15–19]. In addition, TTC3 inhibits neuronal differentiation via RhoA and citron kinase [13]. Research has shown that TTC3 expression is developmentally regulated during human and mouse embryogenesis and becomes restricted to the nervous system at later developmental stages [7, 8]. These results suggest that TTC3 could be an important regulator in neural development and abnormal expression of TTC3 could lead to pathologic phenotypes during embryonic development.

Using the N-terminal 650aa of human TTC3 as bait, we found that DNA polymerase subunit gamma-1 (POLG protein) interacts with TTC3. POLG is the catalytic subunit of DNA polymerase γ (pol γ). In human cells, pol γ is a trimeric protein complex composed of a catalytic subunit of 140 kDa encoded by the *POLG* gene and a dimeric accessory subunit of 55 kDa encoded by the *POLG2* gene (reviewed in Graziewicz *et al.* [20]). Pol γ is the only polymerase found in animal cell mitochondria and is solely responsible for DNA synthesis in all replication, recombination, and repair transactions involving mitochondrial DNA [20, 21]. The *POLG* gene is one of several nuclear genes associated with mitochondrial DNA depletion or deletion disorders [20, 21]. Thus, pol γ supports mitochondrial function.

In neurodegenerative diseases, mitochondrial dysfunction occurs early and contributes to disease pathogenesis (reviewed in Lin and Beal [22]). In addition, misfolding and accumulation of disease proteins characterize numerous late-onset neurodegenerative diseases (reviewed in Douglas and Dillin [23]). Considering the dual effects of TTC3 on mitochondrial functions and protein homeostasis, we speculate that TTC3 may contribute to neurodegenerative pathology, in particular the neurodegenerative phenotypes of DS.

**Figure 6:**
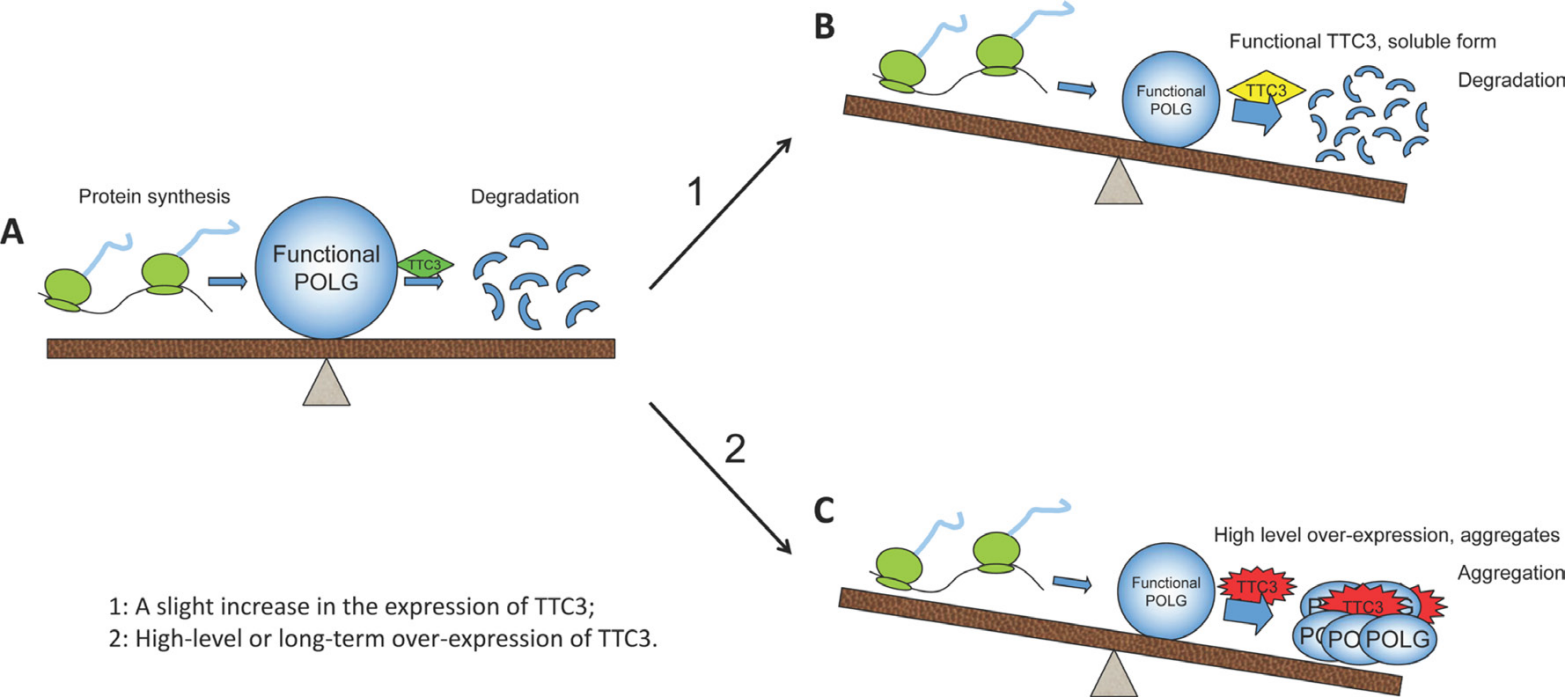
Effects of TTC3 overexpression on functional POLG (**A**) Normally, TTC3 protein is involved in the regulation of POLG stability, and the amount of TTC3 is moderate. The biosynthesis and degradation of POLG protein are maintained in equilibrium by the regulation network. (**B**) Once slightly overexpressed, soluble functional TTC3 facilitates POLG degradation, resulting in a decrease in functional POLG. (**C**) High-level overexpression of TTC3 forms aggregates and interrupts POLG degradation. TTC3 sequesters POLG into the aggregates, resulting in loss of its function.

In this study, we show that soluble TTC3 facilitates POLG degradation, whereas high-level TTC3 overexpression causes aggregates to form and prevents POLG degradation. Accordingly, we propose the following model. Under normal circumstances, the biosynthesis and degradation of POLG protein are in equilibrium. With normal TTC3 protein expression, POLG levels remain relatively constant (Figure 6A). Increasing functional TTC3 levels facilitates poly-ubiquitination of POLG protein and its degradation (Figure 6B). However, high-level TTC3 overexpression slows the degradation of POLG. One possible reason is that, at high levels of overexpression, TTC3 protein tends to be misfolded and form aggregates (shown in Figure 6C). The misfolded TTC3 protein could disturb the functional endogenous TTC3. In addition, excessive TTC3 protein can sequester POLG protein into the aggregates (Figure 2H), which could interfere with the normal function of POLG and interrupt its degradation. Thus, the stability of POLG is influenced by TTC3 in two ways, both of which can result in loss of POLG function. Decreased functional POLG can lead to mitochondrial dysfunction.

As an E3 ligase [9] and a potential co-chaperone [12], TTC3 assists in maintaining protein homeostasis. However, further study is needed to determine whether TTC3 directly ubiquitinates POLG and to delineate the role of the chaperones, such as Hsp70. Based on our results, POLG is not necessarily a substrate for TTC3, but it can be impacted by TTC3. POLG is a catalytic subunit of the only polymerase found in animal cell mitochondria, and the system that regulates POLG degradation is complex and requires further exploration.

## MATERIALS AND METHODS

### Mice

*TTC3*+/– mice were generated using the CRISPR/Cas9 system from the Model Animal Research Center (MARC), Nanjing University. All of the mice in our experiments had C57BL/6J genomic background. All animal welfare and experimental procedures were performed according to guidelines from the Animal Care and Use Committee of MARC.

### Cell culture and transfection

Mouse embryo fibroblasts (MEFs) were derived from wild-type (WT) and *TTC3*+/– mice. HEK293T and MEFs were cultured in Dulbecco’s Modified Eagle’s Medium (DMEM, Thermo Fisher Scientific) supplemented with 10% fetal bovine serum (FBS; Thermo Fisher Scientific) at 37°C in a humidified incubator with 5% CO_2_ (v/v). Neuro2A cells were cultured in DMEM supplemented with 10% FBS and 1% MEM nonessential amino acids (NEAA; Thermo Fisher Scientific) at 37°C in a humidified incubator with 5% CO_2_ (v/v). 293T and N2a cells were transfected by TurboFect Transfection Reagent (Thermo Fisher Scientific).

### Cell lysis

Cells were lysed in RIPA buffer containing 50 mM Tris-HCl (pH 7.6), 150 mM NaCl, 1% NP-40, 0.1% SDS, 0.5% sodium deoxycholate, and 1 × Complete Protease Inhibitor Cocktail (Roche Applied Science). The lysates were clarified by centrifugation at 16,000 × *g* for 20 minutes at 4°C.

Cells were lysed in SDS buffer (1% SDS/PBS) with Roche Complete Protease Inhibitor Cocktail and sonicated. The lysates were clarified by centrifugation at 16,000 × *g* for 20 minutes at room temperature.

### Plasmid constructs

The plasmid mCherry-C1 was a gift from Dr. Zhou Jun, Nankai University, Tianjin, China, and the plasmid pcDNA3.1-HA-Ub was a gift from Dr. Qiu Xiaobo, Beijing Normal University, Beijing, China. Plasmid vector containing a partial cDNA fragment of human TTC3 was purchased from Invitrogen. The 1 to 1478 nt of TTC3 CDS was extended by polymerase chain reaction (PCR) amplification using a pair of primers (TTC3-F1: 5′-CTAGTCGACCATGGACAATTTTGCTGAG; and TTC3-R1: 5′-GCCATCTTGAATTAAGCTTCTCAGC), and the 1479 to 6078 nt of TTC3 was amplified using another pair (TTC3-F1: 5′-GCGCTGAGAAGCTTAATTCAAGATGGC; and TTC3-R2: 5′-GACGGTACCCTACCTAGAAGAGCAGG).

The full-length TTC3 was subcloned into the pCMV-Myc vector (Clontech), using a pair of primers (forward: 5′-TAATAGTCGACATGGACAATTTTGC-3′; and reverse: 5′-AATACGGTACCCTACCTAGAAGAGC-3′) containing Sal I and Kpn I restriction sites. Other forms of TTC3 were generated by the restriction digests or PCR amplifications. The nucleotide sequences in the final constructs used in the study were confirmed.

Plasmid vector containing a partial cDNA fragment of human POLG was purchased from Invitrogen. The full-length POLG was subcloned into the p3xFLAG-CMV10 (Sigma) using a pair of primers (POLG-F1: 5′-CGGAATTCAATGAGCCGCCTGCTC-3′; and POLG-R1: 5′-CGAGATCTCTCCAGGCAGTGTCACTATG-3′) containing EcoR I and Bgl II restriction sites.

### Antibodies

The following antibodies were used: mouse monoclonal anti-c-Myc (9E10; Sigma-Aldrich); mouse monoclonal anti-FLAG (M2; Sigma-Aldrich); mouse monoclonal anti-α-tubulin (DM1A; Sigma-Aldrich); mouse monoclonal anti-HA (HA-7; Sigma-Aldrich); rabbit monoclonal anti-POLG (ab128862; Abcam); rabbit monoclonal anti-POLG (ab128899; Abcam); and rabbit monoclonal anti-POLG (D1Y6R; Cell Signaling).

### Yeast two-hybrid screening

AH109 cells were transformed using the lithium acetate method with the bait plasmid (pGBKT7-Myc-TTC3(1∼650)). Positive expression clones were subsequently mated with Y187 yeast carrying human fetal brain cDNA library. β-Gal–positive clones were identified and sequenced.

### Western blot analysis

Cells were extracted with lysis buffer and then mixed with a one-fifth volume of 5× SDS sample buffer (Genstar, E153). The samples were resolved on SDS-PAGE and transferred to PVDF membranes, and immunoblotting was carried out with antibodies, as indicated, and detected by ECL.

### Live cell imaging

Neuro2A and 293T cells were transfected with indicated plasmids by TurboFect reagents. Image recording was performed at indicated time points using the PerkinElmer UltraVIEW VoX 3D Live Cell Imaging System equipped with an environmental chamber. Cultures were maintained at 37°C and gassed with 5% CO_2_. Images were analyzed with Volocity software.

### Immunoprecipitation

Cells were lysed in RIPA buffer containing protease inhibitors. After clarification, lysates were mixed with the appropriate amount of indicated antibody for 3 hours at 4°C and then mixed with Protein G beads (Dynabeads Protein G; Thermo Fisher Scientific) and incubated for another hour. The beads were subsequently washed three times in lysis buffer and then mixed with 1× SDS sample buffer. Proteins were detected by immunoblotting.

### Ubiquitination assays

For the cellular ubiquitination experiments, cells were transfected with the indicated constructs or control constructs and lysed RIPA buffer with 10 mM *N*-ethylmaleimide (Sigma-Aldrich), immunoprecipitated, and resolved onto SDS-PAGE and immunoblotted.

### Construction of stable cell lines

293T cells were transfected with pcDNA4/TO-myc-TTC3 and pcDNA6/TR plasmid. Stably transfected cells were selected by Zeocin (100 μg/mL) and Blasticidin (10 μg/mL) resistance for 2 weeks. Monoclones were used for experiments.

### CHX assay

293T cells were transfected with indicated plasmids by TurboFect reagents. Twelve hours after transfection, cells were reseeded into four fresh culture dishes with fresh culture medium. Twelve hours later, cells were treated with 100 μg/mL of CHX (at time 0). The cells were harvested at indicated time points and lysed with SDS buffer (1% SDS/PBS) with Roche Protease Inhibitor Cocktail for Western blotting.

For the MEFs, cells were reseeded into three fresh culture dishes with fresh culture medium. Twenty-four hours later, cells were treated with 50 μg/mL of CHX (at time 0). The cells were harvested at indicated time points and lysed with SDS buffer (1% SDS/PBS) with Roche Protease Inhibitor Cocktail for Western blotting.

### Statistical analysis

Data are shown as mean ± standard error. Statistical significance was determined using the Studentʼs *t*-test (two-tailed). Group differences were considered significant at *P* < 0.05.
